# Disulfiram Treatment of Alcoholism

**Published:** 1995

**Authors:** Raymond F. Anton

**Affiliations:** Raymond F. Anton, M.D., is professor of psychiatry and director of alcohol medication studies at the Medical University of South Carolina, Center for Drug and Alcohol Programs, Department of Psychiatry and Behaviorial Sciences, Charleston, South Carolina

**Keywords:** disulfiram, drug therapy, AOD dependence, drug efficacy, veteran

**Figure:**
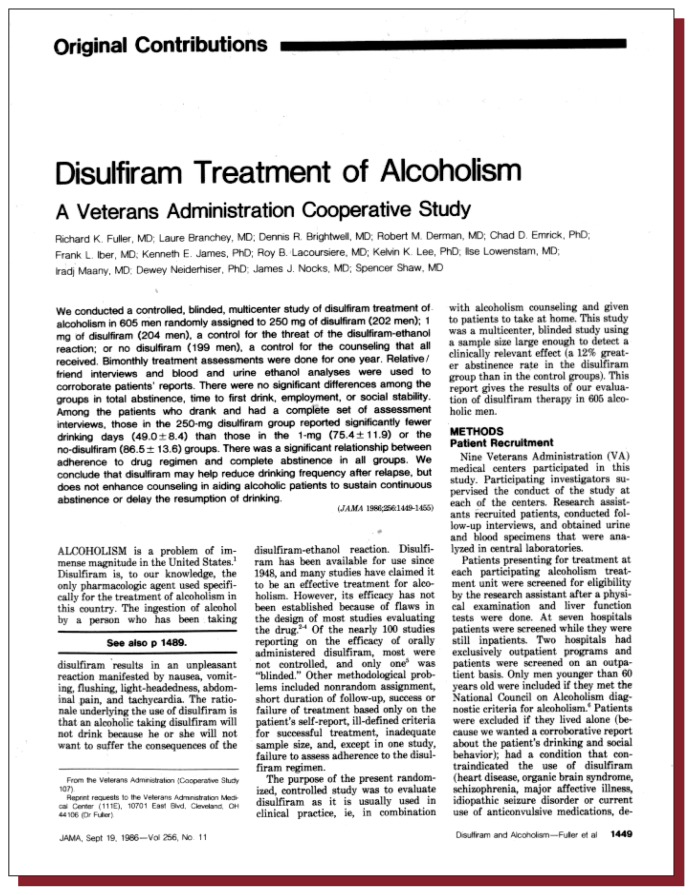
Fuller, R.K.; Branchey, L.; Brightwell, D.R.; Derman, R.M.; Emrick, C.D.; Iber, F.L.; James, K.E.; Lacoursiere, R.B.; Lee, K.K.; Lowenstam, I.; Maany, I.; Neiderhiser, D.; Nocks, J.J.; and Shaw, S. Disulfiram treatment of alcoholism: A Veterans Administration cooperative study. *Journal of the American Medical Association* 256(11):1449–1455, 1986.

Previous to the completion of this well-designed scientific study, which was conducted at nine Veterans Administration Medical Centers, the medication disulfiram (Antabuse^®^) was controversial in the treatment of alcoholism. The controversy over its efficacy was rooted in the lack of sophisticated scientific data on which to base its effectiveness. Fuller and colleagues used state-of-the-art clinical research methods to design and conduct a study that could answer the question of disulfiram’s efficacy beyond a reasonable doubt.

The study reported in this article not only provided answers to important questions regarding the use of disulfiram in alcoholism treatment but also defined the methodology for conducting this type of research. It has served as a model for testing the usefulness of medication treatment of alcoholism for the past 10 years. For instance, Fuller and colleagues gave much attention to patient selection criteria; the use of placebo (i.e., inactive) pills for comparison; validation of patient self-reports of drinking, both with the use of reports from cohabiting friends or relatives (collaborative sources) and biological measurements; and medication compliance markers in the urine. In addition, Fuller and colleagues also emphasized new statistical methods (i.e., survival analysis, which utilizes the time it takes to relapse to alcohol use as the primary unit of analysis), which have been used successfully in other branches of health care research, into the alcoholism treatment research arena.

In this year-long study, 605 male veterans were assigned at random to three medication treatment groups: active disulfiram (i.e., 250-milligram dose), inactive disulfiram (i.e., 1-milligram dose), and placebo (i.e., no dose). Patients were expected to attend supportive counseling sessions during the course of the study and were encouraged, but not mandated, to attend Alcoholics Anonymous meetings as well. Each patient and his collaborative source were questioned periodically during the study about the patient’s alcohol consumption and social well-being. The interviewers were unaware of the patient’s medication group assignment. Fuller and colleagues used this information to calculate abstinence rates and the time elapsed prior to a return to drinking for subjects in the three medication treatment groups.

Fuller and colleagues sum up their main finding as follows:

Using a randomized, controlled, blinded study design, we did not find that disulfiram provided additional benefit to the treatment services provided at our nine clinics in aiding our patients to remain completely abstinent or in delaying the time to relapse (p. 1454).

A positive finding, however, was that patients who were given disulfiram had fewer drinking days during the study when compared with the patients in the other medication groups. These patients were slightly older, had been alcohol abusers longer, and had lived at their current addresses longer than had those who relapsed. Fuller and colleagues go on to say,

Thus, the results of this study indicate that disulfiram is not necessary for those patients able to achieve total abstinence [about 20 percent of the total number of patients entering the study] but suggest that disulfiram be reserved for those older, more socially stable men who relapse (p. 1454).

As the authors suggest in their concluding remarks, the generalizability of the results may be limited because the population under study did not include subjects of higher socioeconomic employment or women. However, this landmark study indicated that well-founded scientific inquiry could be applied to alcoholism treatment to achieve clinically useful results.

Despite the rather straightforward and clear results of this investigation, some clinicians and researchers could not completely accept the overall findings because they could point to patients who successfully used disulfiram to achieve long-lasting sobriety. This led investigators to inquire about the conditions under which disulfiram could be used with a greater expectation of success. For example, Chick and colleagues (1992) studied patients whose disulfiram intake was monitored and found that patients who took disulfiram under controlled circumstances did better than those who did not.

Since the publication of this seminal article by Fuller and colleagues, other studies of disulfiram as well as related aversive treatment medications all seem to point to motivation and compliance as crucial variables that may predict who will respond best to this type of treatment approach (e.g., Allen and Litten 1992).

In many ways, this seminal study proved to be a watershed for future investigation of pharmacologic agents for the treatment of alcoholism. It paved a methodologic pathway for other treatment outcome studies to follow. For example, a large Veterans Administration cooperative study on the efficacy of lithium carbonate in alcoholics borrowed heavily from the methodologies developed in Fuller and colleagues’ disulfiram trial (Dorus et al. 1989). Defined patient-selection criteria, compliance monitoring, validation of patient drinking reports, and survival analyses all continue to be mainstays of modern alcoholism treatment research.

The technological “spinoffs” from this thoughtful scientific endeavor are beginning to pay off as the discovery of new medications for the treatment of alcoholism are coming to fruition. For this seminal alcoholism research study, the future then, is now.
